# Deciphering Transcriptional Programming during Pod and Seed Development Using RNA-Seq in Pigeonpea (*Cajanus cajan*)

**DOI:** 10.1371/journal.pone.0164959

**Published:** 2016-10-19

**Authors:** Lekha T. Pazhamala, Gaurav Agarwal, Prasad Bajaj, Vinay Kumar, Akanksha Kulshreshtha, Rachit K. Saxena, Rajeev K. Varshney

**Affiliations:** 1 International Crops Research Institute for the Semi-Arid Tropics (ICRISAT), Hyderabad, 502 324, India; 2 School of Plant Biology and Institute of Agriculture, The University of Western Australia, 35 Stirling Highway, Crawley, WA, 6009, Australia; National Institute for Plant Genome Research, INDIA

## Abstract

Seed development is an important event in plant life cycle that has interested humankind since ages, especially in crops of economic importance. Pigeonpea is an important grain legume of the semi-arid tropics, used mainly for its protein rich seeds. In order to understand the transcriptional programming during the pod and seed development, RNA-seq data was generated from embryo sac from the day of anthesis (0 DAA), seed and pod wall (5, 10, 20 and 30 DAA) of pigeonpea variety “Asha” (ICPL 87119) using Illumina HiSeq 2500. About 684 million sequencing reads have been generated from nine samples, which resulted in the identification of 27,441 expressed genes after sequence analysis. These genes have been studied for their differentially expression, co-expression, temporal and spatial gene expression. We have also used the RNA-seq data to identify important seed-specific transcription factors, biological processes and associated pathways during seed development process in pigeonpea. The comprehensive gene expression study from flowering to mature pod development in pigeonpea would be crucial in identifying candidate genes involved in seed traits directly or indirectly related to yield and quality. The dataset will serve as an important resource for gene discovery and deciphering the molecular mechanisms underlying various seed related traits.

## Introduction

Pigeonpea is an important grain legume in the semi-arid regions of Asia and Africa where it plays an important role in human nutrition and soil health. Pigeonpea seeds are rich sources of proteins, carbohydrates, fibers and micronutrients such as iron, selenium, magnesium, calcium, phosphorus, potassium etc. Legume researchers working in pigeonpea improvement are striving to understand the genetic control related to seed development, seed protein content, pod filling, grain weight etc. Genomics-assisted breeding can greatly accelerate these efforts by identifying the candidate genes and the genomic regions for targeted traits [1, 2]. Majority of the basic genetic studies of seed development have been carried out in model plants such as Arabidopsis [3, 4]. Such studies are lacking in the case of pigeonpea. In recent years, RNA-seq has emerged as a powerful tool for measuring the levels of transcripts of the entire transcriptome and evaluating differential expression more precisely than other techniques such as microarrays etc. [5–6]. Thus, RNA-seq has increasingly been used for understanding the developmental processes such as fruit ripening, flowering, seed, embryo and endosperm development [7–12].

In view of the above, we have used pigeonpea variety, ‘Asha’ (ICPL 87119) for studying seed development in pigeonpea. Asha is a widely cultivated variety, resistant to a number of stresses and has been used for the development of draft genome assembly [13]. Here, we scrutinize the spatio-temporal gene expression during pod and seed development involving organization of embryo tissues, cell differentiation, signal transduction followed by accumulation of storage compounds and seed desiccation. These are highly regulated, metabolically active processes associated with temporally distinct metabolic switches [14]. Transcriptome profiling was carried out to understand this programming and re-programming from flowering to mature pod formation. Any defect in this programming could be exemplified into a severe defect in the grain quality as well as yield in total. The knowledge could be utilized for improving the genetic resource and seed quality traits in pigeonpea.

## Materials and Methods

### Plant material and sample preparation

The pigeonpea variety, ICPL 87119 (Asha) was grown under glasshouse conditions in three biological replications. About 50–100 buds were tagged with different colored threads representing the date of tagging for harvesting embryo sac (Em), seed (S) and pod wall (Pw). Flowers and young to mature pods were sampled at 0, 5, 10, 20 and 30 days after anthesis (DAA), respectively. In the present study, DAA was determined by the day on which flower is completely opened and petals fully extended. From each tagged set, embryo sac was excised from the flowers (0 DAA), while seeds and pod walls were dissected from the pods collected at 5, 10, 20 and 30 DAA. Care was taken to harvest pods of the same size measured by a ruler for a particular time point. The samples are represented throughout the manuscript as tissue_stage, where tissue type is abbreviated (Em for embryo sac, Sd for seed and Pw for pod wall) followed by an underscore and the stage (0, 5, 10, 20 and 30 DAA). Smaller tissues such as embryo sac (Em_0), seed at 5 DAA (Sd_5) and pod wall at 5 DAA (Pw_5) were dissected using a stereomicroscope (Olympus, Tokyo) followed by flash-freezing in liquid nitrogen and stored at -80°C until RNA isolation.

Total RNA was isolated from above mentioned nine tissues (S1 Fig), using Ambion RNAqueous^®^-Micro kit (AM1913, Ambion, USA) according to manufacturer's instructions. The quality of total RNA was assessed using Agilent RNA 6000 Nano chip on Agilent 2100 Bioanalyzer (Agilent Technologies, Palo Alto, CA, USA). RNA samples with RNA integrity value of > 8.0 were included for the study and quantified using Qubit^™^ 2.0 Fluorometer (Thermo Fisher Scientific Inc., USA) with Qubit^™^ RNA Assay Kit (Thermo Fisher Scientific Inc., USA).

### RNA sequencing and data pre-processing

Total RNA from three biological replicates of each sample was pooled in equimolar concentration prior to library preparation. Approximately 2.5 μg of total RNA was used for library preparation following Illumina TruSeq RNA Sample Preparation v2 LS Kit (Illumina Inc., San Diego, CA) according to manufacturer’s instructions. The quantification and size distribution of the enriched libraries were checked using Agilent 2100 Bioanalyzer system (Agilent Biotechnologies, Palo Alto, USA) with High Sensitivity DNA kit. All libraries were sequenced on Illumina HiSeq 2500 platform to generate 100 base paired-end reads. The raw reads were subjected to quality filtering using Trimmomatic v0.35 [15] to remove low quality sequencing reads and any adapter contamination.

### Global and differential gene expression analysis

The RNA-seq data was analyzed using the “Tuxedo” pipeline [16]. TopHat v2.1.0 [17] and Bowtie v2.2.5 [18] was used to align and map the reads of all nine samples on the pigeonpea reference genome [13]. The alignment file from tophat2 for each sample along with reference genome GFF was used to perform RABT (reference annotation based transcript) assembly through Cufflinks v2.2.1 [16, 19] to assemble genes and isoforms. These cufflink assemblies were then compared and merged using cuffmerge script from cufflinks to remove transfrags and generate a combined GTF for further downstream analysis. Further, cuffdiff [20] was used to identify differentially expressed genes (DEGs).

### Gene clustering and visualization

K-means clustering algorithm was used to visualize the genes exhibiting a similar expression pattern. K-means clustering was performed on log_2_ transformed FPKM values using MeV v4.8.1 [21] with Pearson’s correlation as similarity metrics. To determine optimal number of clusters, sum of squared error values were plotted against the different values of K ranging from 2 to 15 [22].

### Gene annotations, GO term and pathway assignment

Genes were subjected to BLASTX similarity search against NCBI non-redundant (nr) Viridiplantae protein database with cut-off of E-value ≤10−5, to identify significant hits. These blastx results were then used to identify Gene Ontology (GO) annotation and pathways through Blast2GO v3.3 [23]. Further, transcription factor encoding genes were identified by aligning sequences against PlantTFDB 3.0 [24] (E-value ≤10−5). Identification of tissue-specific genes was performed on genes with FPKM ≥ 2 in at least one of nine samples, by calculating Tissue specificity index (τ) [25] using the equation:

$$\tau =\frac{\sum_{\text{i}=1}^{\text{N}}\left(1-{\text{x}}_{\text{i}}\right)}{\text{N}-1}$$

where, N is the number of samples and x_i_ is the expression value of a gene normalized by maximum value across all samples. The value of τ range from 0 to 1, where higher the value more likely the gene is specifically expressed in that stage. For this study, genes with τ ≥ 0.9 were considered as tissue specific.

### Real-time quantitative polymerase chain reaction

Real-time quantitative polymerase chain reaction (qPCR) was carried out using Applied Biosystems 7500 Real Time PCR System with the SYBR green chemistry (Applied Biosystems, USA). The gene specific primers were designed using PrimerQuest (Integrated DNA Technologies, http://www.idtdna.com) with default parameters for qPCR (S5 Table). qPCR reactions were performed using SYBR green master-mix in 96 well plates with two technical replicates and two biological replicates using Actin as an endogenous control. PCR conditions included a pre-incubation for 2 min at 50°C, denaturation for 10 min at 95°C, followed by 40 cycles of denaturation at 95°C for 15s and 1 min at 60°C for annealing and extension. Efficiency of amplification for all the primers were assessed which ranged from 95% to 101%, while melting curve analysis was performed to determine the specificity of the reaction. The correlation between the qPCR data and the RNA-seq data was established using Microsoft Excel 2010.

## Results

### Global overview of gene expression

Analysis of the transcriptome profiles of embryo sac, seed and pod wall was conducted to study the seed and pod development in pigeonpea. In order to do so, we have selected seed and pod wall from four stages (5, 10, 20 and 30 DAA) along with embryo sac from 0 DAA for transcriptome profiling. RNA samples from embryo sac (Em_0), seed (Sd_5, Sd_10, Sd_20 and Sd_30) and pod wall (Pw_5, Pw_10, Pw_20 and Pw_30) were sequenced (S1 Fig). About 684 million sequencing reads were generated and after the quality filtration, 632 million reads (92.5% of the total reads) were finally used for downstream analyses. All raw sequencing data have been deposited in NCBI Sequence Read Archive (SRA) database with the BioProject ID-PRJNA344973. On an average, 95.8% of the high quality reads could be mapped onto the pigeonpea reference genome ranging from 92% in Sd_20 to 96.8% in Sd_10 (Table 1).

**Table 1:** Summary statistics for Illumina sequencing data and mapping to the pigeonpea genome.

Tissues	Days after flowering (DAA)	Raw reads	Filtered reads	Mapped	Mapping rate
Embryo sac	0	88,258,170	81,488,665	78,735,124	96.62%
Seed	5	72,892,594	66,891,663	64,421,509	96.31%
Pod shell		68,166,862	62,886,396	60,819,898	96.71%
Seed	10	82,806,502	76,881,629	74,386,738	96.75%
Pod shell		68,838,304	63,840,679	61,447,041	96.25%
Seed	20	94,557,904	87,896,111	80,898,608	92.04%
Pod shell		79,700,848	74,196,201	71,391,941	96.22%
Seed	30	58,976,156	53,835,089	51,424,473	95.52%
Pod shell		69,799,824	64,959,502	62,414,455	96.08%
Total		683,997,164	632,875,935	605,939,787	

**Fig 1:**
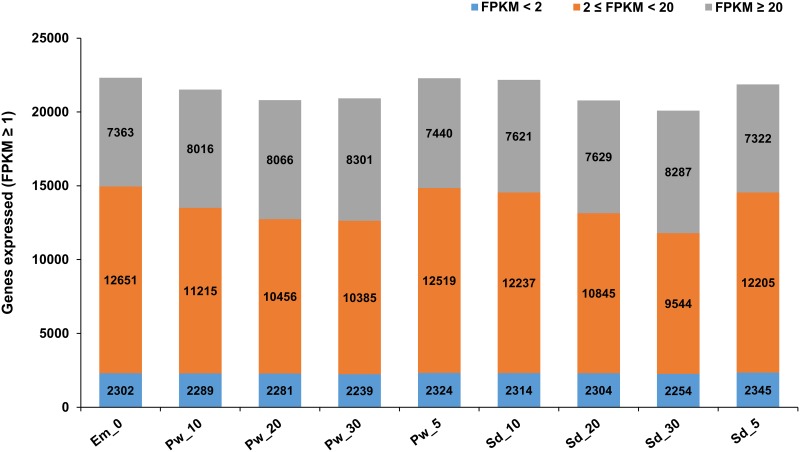
Distribution of genes expressed in embryo sac, seed and pod wall samples. Genes were grouped into three categories based on expression level (FPKM<2, 2 ≤ FPKM < 20 and FPKM ≥20) in nine samples. These include embryo sac (0 DAA), seed and pod wall from four different developmental stages (5, 10, 20, 30 DAA).

**Fig 2:**
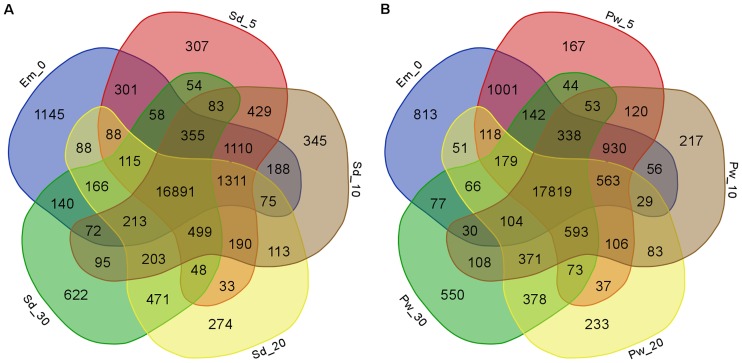
Commonly and specifically expressed genes in embryo sac, seed and pod wall samples. Venn diagram depicting the commonly and specifically expressed genes in samples Em_0, Sd_5 to Sd_30 (A) and in samples Em_0, Pw_5 to Pw_30 (B). Numbers depict the number of genes expressed either specifically in one tissue (individual section/color) or commonly among different tissues (overlapping sections/color) in the Venn diagram.

A gene was considered to be expressed in a sample if FPKM ≥ 1(Fragments Per Kilobase Of Exon Per Million Fragments Mapped) and quantification status as ‘OK’ leading to total 27,441 expressed gene loci in nine samples. Further, based on the expression levels, we categorized them into three major categories, FPKM<2, 2 ≤ FPKM < 20 and FPKM ≥20. We defined the moderate expression to those in the category 2 ≤ FPKM < 20, whereas FPKM<2 and FPKM ≥20 as low and highly expressed genes, respectively. Distribution of genes expressed in nine samples under these categorization based on their expression levels (low, moderate and high) has been provided in Fig 1. Among the nine samples, Em_0 followed by Pw_5, Sd_10, and Sd_5 had the maximum number of expressed genes. These samples represent the early stages of seed and pod wall development. On the other hand, the largest number of highly expressed genes (FPKM ≥20) was observed in the later stages of seed (Sd_30) and pod wall (Pw_30) development with 8,287 and 8,301 genes, respectively. Among the total 27,441 genes identified, a set of 22,316 genes were expressed in Em_0, whereas 24,937and 24,636 in seed and pod wall, respectively including the four developmental stages (Fig 2A and 2B). A total of 16,891 genes were expressed commonly in all the four developmental stages of seed including the embryo sac. Similarly, 17,819 genes were expressed commonly in embryo sac and the four developmental stages of pod wall. A set of 627 genes was further identified to be constitutively and stably expressed with CV (coefficient of variation) ranging from 5 to 15% in all the samples, including embryo sac, seed and pod wall throughout the four time points. The top constitutively expressed genes with CV (coefficient of variation) ranging from 5.33 to 5.89% includes flowering time control FCA isoform X1, probable NOT transcription complex subunit VIP2 isoform X3, C-terminal binding AN, armadillo repeat-containing LFR-like, FAR1-RELATED SEQUENCE 5 (S1 Table).

**Fig 3:**
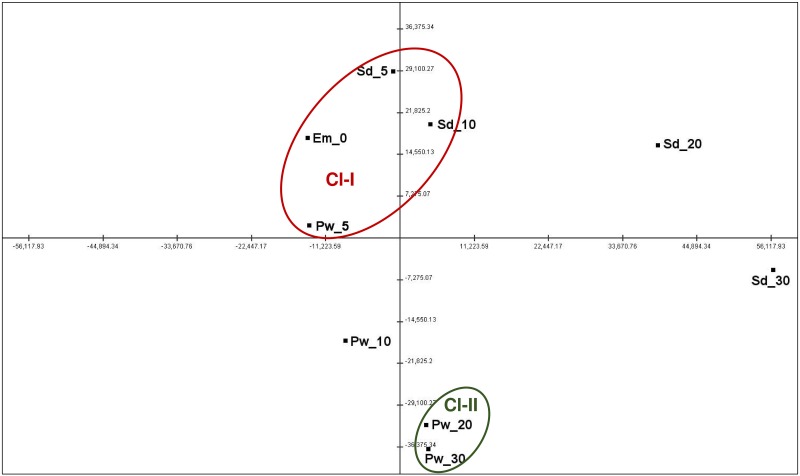
Principal component analysis depicting correlation among samples based on gene expression data. Samples formed two clusters representing early stage of seed development (Cl-I) and late pod wall development (Cl-II). Red color depicts the cluster of samples from the early stage of seed development, while green color depicts cluster of sample that represents pod wall development.

Furthermore, principle component analysis (PCA) was performed to analyze the relationships among the samples based on their expression values (Fig 3). PCA has clustered the nine samples into two major clusters, namely Cl-I and Cl-II. These clusters represented early stages of seed and pod wall development (Cl-I), whereas Cl-II represented late pod wall development. Cl-I consisted of samples, Em_0, Sd_5, Sd_10 and Pw_5 while Cl-II were composed of two pod wall samples, Pw_20 and Pw_30. Samples Pw_10, Sd_20 and Sd_30 could not be assigned to any of the clusters, representing different stages of development and growth. Sample Sd_30 represented seed maturity phase, while Pw_10, Sd_20 reflected the intermediate stage of development, between the early and late developmental phases.

#### GO annotations and pathways

**Fig 4:**
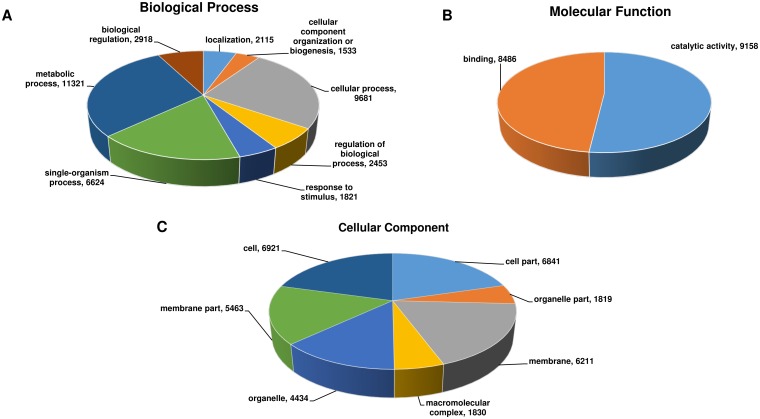
Distribution of Gene Ontology annotation assigned by Blast2GO. Summary of level 2 GO annotation into three categories, biological processes (A), molecular functions (B), and cellular components (C) are represented in a pie-chart.

The putative functional annotation and GO terms were assigned to 27,441 genes using NCBI non-redundant (nr) Viridiplantae protein database. Among 27,441, 17,700 genes accounting for ~92% were assigned GO term for biological processes, molecular functions and cellular components (Fig 4). These genes were found to be involved in biological processes such as metabolic (63.96%) and cellular (54.69%) processes, while molecular functions included catalytic and binding activities (51.74%). Among the cellular components, cell, membrane and organelles were the most predominant ones. Additionally, 9,309 genes could be assigned and mapped to 145 different pathways in KEGG database [26]. Majority of the genes were mapped to the pathways related to purine metabolism (1149), thiamine metabolism (920), biosynthesis of antibiotics (498), starch and sucrose metabolism (413). The complete list of pathways that were assigned to genes has been provided in S2 Table. Among 27,441 genes, 11,243 were identified as encoding transcription factors (TFs) categorized into 58 TF families. Majority of these TFs were categorized into bHLH (1141), NAC (754), MYB-related (635), WRKY (591) among others (S3 Table).

### Differentially expressed genes

**Fig 5:**
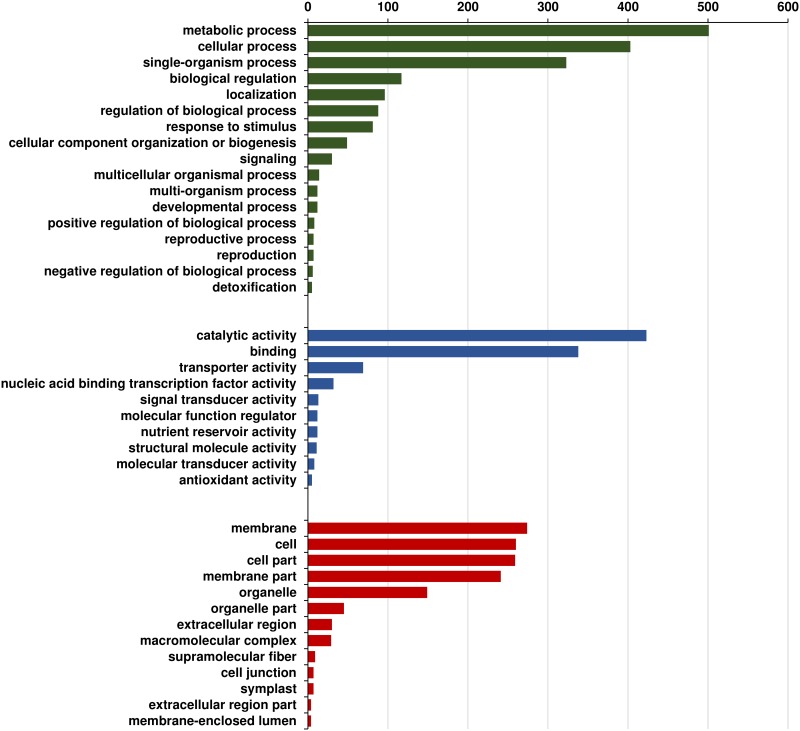
Gene Ontology annotation of differentially expressed genes. Bar graph representing level 2 GO annotation into three categories, biological processes (A), molecular functions (B), and cellular components (C).

Expression profiles generated in nine samples were compared to study “spatio-temporal” gene expression. In order to do so, 12 pair-wise comparisons were made among different samples harvested at four different time points to identify DEGs. We have compared samples to their successive stages to identify DEGs at different time points in seed and pod wall separately. This includes Em_0 with Sd_5, Sd_5 with Sd_10, Sd_10 with Sd_20 and Sd_20 with Sd_30 for studying seed development. Similarly, comparisons were made between Em_0 with Pw_5, Pw_5 with Pw_10, Pw_10 with Pw_20 and Pw20 with Pw_30. Likewise, four pair-wise comparisons were also made between seed and pod wall samples (Sd_5 with Pw_5, Sd_10 with Pw_10, Sd_20 with Pw_20 and Sd_30 with Pw_30) at a particular stage to study spatial expression. As a result of above mentioned pairwise comparisons, a total of 7,415 DEGs were finally identified with 3,557 and 3,858 induced and repressed, respectively in different pairwise comparisons (S4 Table). GO annotation of these DEGs identified major biological processes such as metabolic, cellular, single-organism processes, biological regulation and localization (Fig 5). In order to validate the RNA-seq data, 18 genes were randomly selected for qPCR analysis. A correlation coefficient of 0.71 have been found between the RNA-seq data (log_2_-fold change) and qPCR (log_2_-fold change) values (S2 Fig, S5 Table). These included genes encoding Protein LEAFY COTYLEDON 1-LIKE, sugar transporter sweet1-like, expansin-like B1, TGACG-sequence-specific DNA-binding TGA- partial which showed high-degree of correlation between RNA-seq and qPCR data.

#### Temporal gene expression

**Fig 6:**
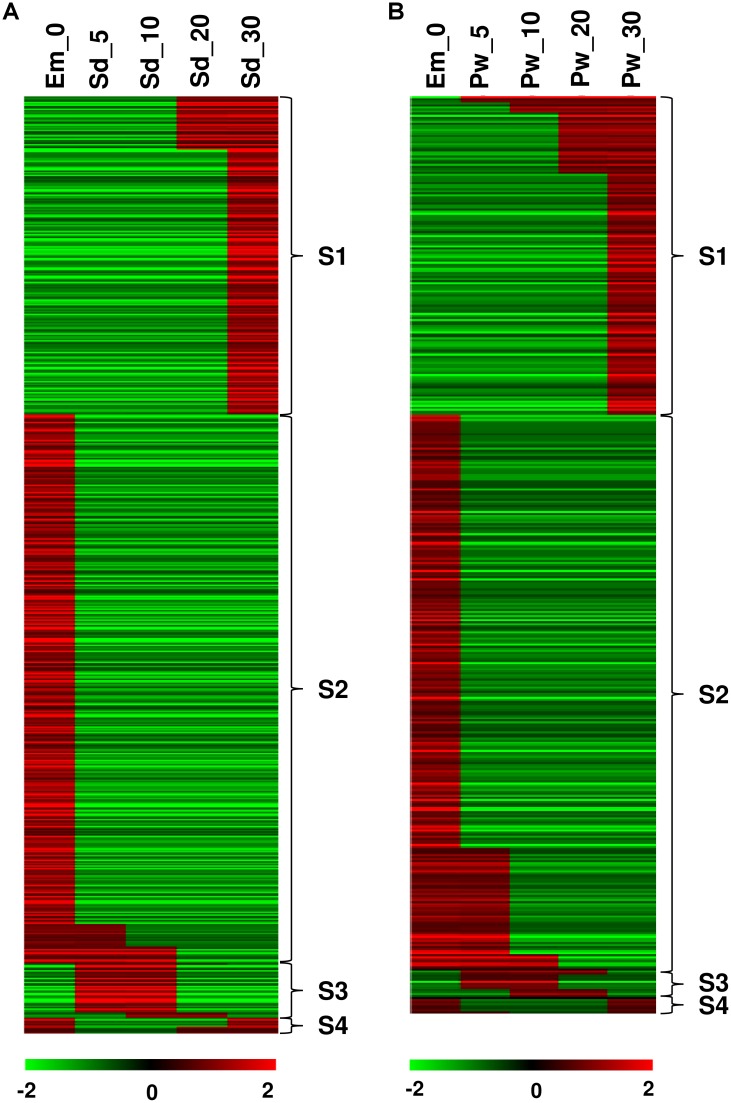
Temporal expression patterns in seed and pod wall. Figure depicts temporal patterns of gene expression in (A) developing seed from Em_0 to Sd_30 and in (B) developing pod wall from Em_0 to Pw_30. The transitions in the pattern of gene expression were studied using StepMiner algorithm. Between two consecutive time points, four different transitions were identified, namely one-step-up, one-step-down, two-step-up-down and two-step-down-up transitions which were referred as S1, S2, S3 and S4, respectively. Red color depicts up-regulation and the green color shows down-regulation.

To understand the well-orchestrated processes involved in transition during seed/pod wall development from embryo sac, we studied temporal patterns of gene expression in the developing seed and pod wall spanning 0 to 30 DAA. Temporal expression pattern was characterized using StepMiner algorithm [27] that identified one or two transition points in expression. We have analyzed the data in two sets, seed (Fig 6A, S6 Table) and pod wall (Fig 6B, S7 Table) developmental transition from 0 to 30 DAA, separately. The analysis have identified 668 and 449 genes in seed and pod wall, respectively that showed four temporal gene expression patterns. These temporal patterns of gene expression were referred as S1, S2, S3 and S4, depicting one-step-up, one-step-down, two-step-up-down and two-step-down-up transitions in two consecutive time points.

In the case of seed development, one-step transition identified the maximum number of genes with 390 and 227 genes showing one-step-down (expression from high to low) and one-step-up (expression from low to high) transition, respectively (Fig 6A). One-step-down transition was involved during 0 to 30 DAA, that means a set of 390 genes were down regulated in seed samples during 5 to 30 DAA, which were otherwise up-regulated in the Em_0 sample. Similarly, in the one-step-up transition, 227 genes were identified as down regulated in the early stage of seed development (Em_0, Sd_5, Sd_10), but up-regulated during seed maturity stage (Sd_20 and Sd_30). In both the transitions, gene involved in defense and signal transduction were fairly evident with biological processes such as peptidyl-tyrosine phosphorylation, oxidation-reduction process, cell wall modification/organization (S6 and S7 Tables). During two-step-up-down and two-step-down-up transitions, 39 and 11 genes were identified, respectively. Two-step-up-down transition identified genes that were initially down-regulated in embryo sac (Em_0), followed by an up-regulation in immature seed samples (Sd_5 and Sd_10) and then down regulation in the mature seed samples (20 and 30 DAA). In two-step-down-up transition, the genes were up-regulated in Em_0, Sd_ 20 and Sd_30, but down regulated in Sd_5 and Sd_10. During two-step transition, genes related to signal transduction and defense seems conspicuous with genes encoding serine threonine-kinase At5g01020-like isoform X1, probable calcium-binding CML22 etc.

The temporal gene expression pattern in the case of pod wall development was found to have similar pattern of expression to that of the seed development (Fig 6B). One-step-down (expression from high to low) and one-step-up (expression from low to high) transition identified 272 and 156 genes, respectively in pod wall samples. One-step-down transition involved genes encoding transcription factors and enzymes related to cell wall metabolism, whereas one-step-up involved mainly signal transduction and defense. Two-step-up-down and two-step-down-up transitions identified 13 and 8 genes, respectively which were involved in the biosynthesis of secondary metabolites and defense.

#### Spatial gene expression

**Fig 7:**
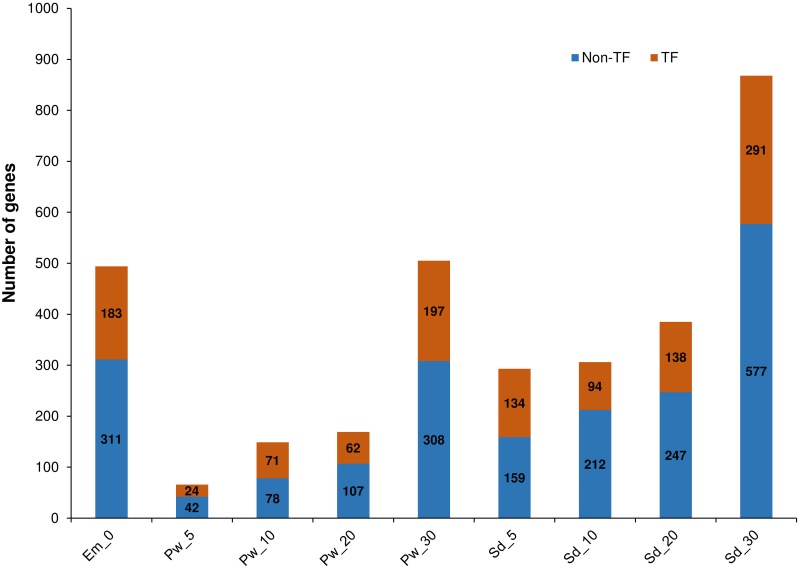
Tissue-specific genes identified in nine samples. Figure depicts the number of genes identified specifically in the nine samples. Tissue specific genes were identified by calculating Tissue specificity index (τ). Genes with τ ≥ 0.9 were considered as tissue specific in our present study. Orange color represents the proportion of genes encoding transcription factors (TF) and blue color represents the proportion of genes that encodes proteins other than transcription factors (Non-TF).

Tissue-specific expression was exhibited by 3,235 genes in nine samples, which included TF (1194) and non-TF (2041) encoding genes (Fig 7, S8 Table). Highest number of specifically expressed genes were identified in samples Em_0 (494 genes), Sd_30 (868 genes) and Pw_30 (505 genes), which included 37 to 39% of TF encoding genes. Among the nine samples, Sd_5 and Pw_10 expressed a higher proportion of the TF encoding genes, about 46 and 48%, respectively. TFs predominant in Sd_5 include 13 B3 domain-containing and 15 C2H2 zinc-finger, whereas in Pw_10, 11 ERF and 12 MYB and MYB-related TFs were involved. In embryo sac, basic helix-loop-helix (bHLH) DNA-binding superfamily protein was identified as the largest family of TFs that were expressed, followed by NAC, FAR1, MYB and WRKY. In mature seed Sd_30, bHLH, ERF and HSF were identified as the most expressed family of TFs.

### Gene clustering

**Fig 8:**
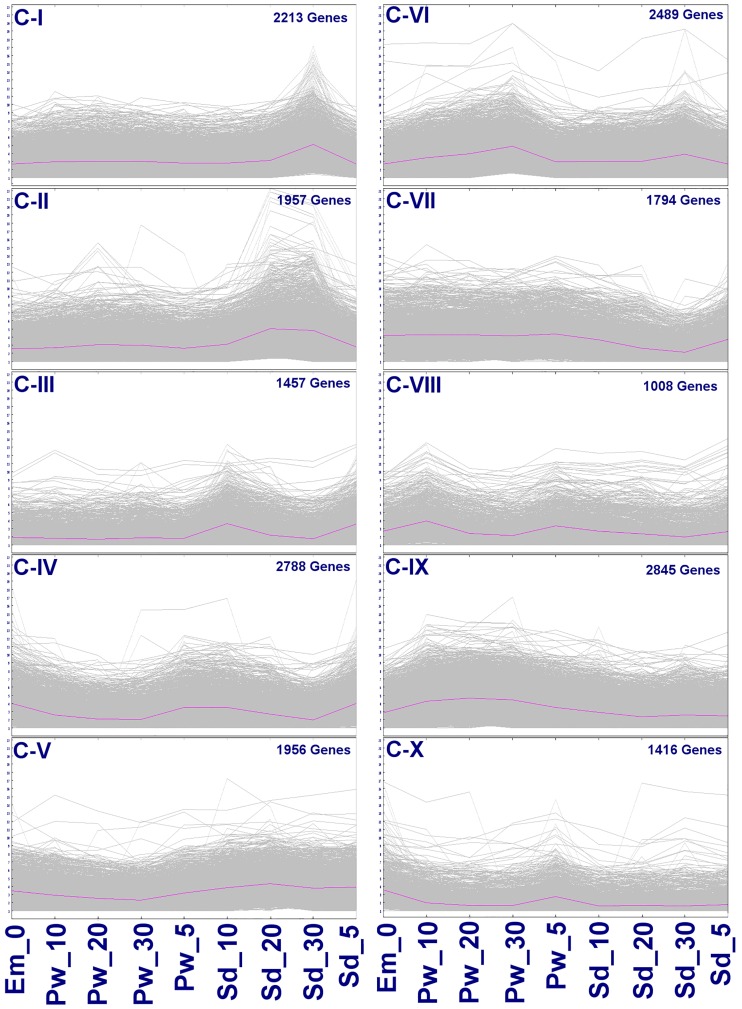
Gene clusters identified in nine samples. Ten gene clusters (C-I to C-X) were identified using k-means clustering. The x-axis represents the samples, while the y-axis represents log_2_ transformed FPKM derived from RNA-seq data for each of the samples. The genes were clustered based on similarity of expression pattern during the course of development.

K-means clustering is useful for studying gene interactions especially when the genes either are regulating or are being regulated for specific biological process. This analysis allows measuring the dynamic expression of genes during a time-series comprising of different tissues in different developmental phases. K-means clustering generated ten gene clusters based on their similar expression pattern (Fig 8). In these clusters, genes ranging from 1008 to 2845 formed co-expressed groups, with C-IX composed of most number of genes (2845) while C-VIII consisted of least number of the genes (1008). GO annotation of these genes revealed metabolic processes, oxidation-reduction processes, protein phosphorylation, defense and cell wall biogenesis/modification related activities in all the clusters. Besides these activities, specialized activities were also evident in few clusters. C-III and IV were found to be involved during early seed development represented by seed samples, Sd_5 and Sd_10, mainly associated with storage protein (stem 28 kDa glycoprotein), cell wall biogenesis, organization and modification (expansin B1, B2, A22). C-IV also composed of genes that were expressed in samples Em_0, Pw_5, Sd_5 and Sd_10, mainly with cell cycle related activities, such as asymmetric cell division, radial pattern formation, negative regulation of mitotic cell cycle etc. Clusters, C-VII and C-IX showed higher expressions in all the samples (Em_0, Pw_5 to 30) except Sd_20 and Sd_30, which displayed slightly lower expression. In these clusters, activities such as photosynthesis, response to light stimuli, cell wall biogenesis, organization and modification, flavonoid biosynthesis were primarily involved in the pod wall. C-VIII and C-X showed distinct expression in Em_0 and Pw_5, which were involved in cell wall biogenesis/modification and auxin-activated signaling pathway. C-I, II, V and VI showed higher expression of genes during Sd_30 involved mainly in seed maturity (seed maturation LEA 4, PM35, PM41, seed dormancy control), cotyledon development (CUP-SHAPED COTYLEDON 2), dehiscence and nutrient reservoir activity (glycinin G3, glutelin type-A 2). C-II and V gene clusters were also found to have higher expression during Sd_20. C-II was composed of genes encoding TF containing B3 DNA binding domain (ABI3, FUS3, NGA1, VAL1-like), cell division cycle 48 homolog, 123 homolog, cell number regulator 6-like, cell division cycle 48 homolog, whereas C-V were composed of genes encoding cell cycle checkpoint related proteins, such as RAD1 and C-VI were mainly related to kinase activity.

In this way, four groups of co-expressed genes were revealed in this study. The first group of co-expressed gene cluster (C-I, II, V and VI) included those that showed higher levels of expression in mature tissues but lower levels of expression in immature tissues. These genes seem to be predominantly involved in seed maturation, desiccation and stress response. The second group of co-expressed gene clusters (C-III, IV, VII and IX) included those which showed higher expression in immature tissues but lower expression in mature tissues. These genes seemed to be associated primarily with storage protein accumulation, photosynthesis, cell wall biogenesis/ modification, flavonoid biosynthesis, cell cycle and cell division. The third group of gene cluster (C-VIII and X) composed of the genes involved mainly in early differentiation from Em_0 to Pw_5.

## Discussion

Pigeonpea seeds are rich source of protein (20–23%) and form an ideal combination with cereals for a balanced diet. Seed development is perhaps one of the most regulated and metabolically active events in the plant life cycle. Understanding the transcriptional programming involved in regulated and metabolically active process of seeds development in pigeonpea would be a crucial step. The role of metabolic and hormonal regulation in legume seed development involving biosynthetic fluxes and assimilates partitioning has been previously studied. The changing oxygen, energy and nutrient status have profound influence at the transcriptional and protein levels during seed development [3]. Complementing these finding, we studied spatial and temporal expression during seed and pod development in pigeonpea using RNA-seq. In addition to seeds, pod wall was also studied over the course of development as the latter have many important implication with contribution to seed grain filling [28].

Seed development in angiosperm is considered to be composed of phases which involves cell division and expansion, nutrient accumulation and seed moisture loss [29]. Temporal gene expression in the seeds and pod wall development were studied roughly during these phases, 5, 10, 20 and 30 DAA. The sample of 0 and 5 DAA reflects more or less the cell division and expansion phase which seems mostly governed by auxin signaling [30], followed by an active nutrient reserve accumulation associated with high-energy generating processes taking place during 10 and 20 DAA. To recognize the physiological maturity in legume seeds, the identified parameters seem to be when the pods lose their bright green color [31], considering this, we collected seed and pod samples at 30 DAA to represent the mature seed/ pod. During seed maturity, activities related to seed storage proteins accumulation, desiccation tolerance and pathogen defense were more pronounced. In contrast, pod wall development mainly involves cell wall modification, accumulation of defensin, fatty acid and secondary metabolite biosynthesis. Interestingly, during pod wall development from 10 to 20 DAA, genes encoding pro-glycinin and sucrose binding protein were induced, responsible for nutrient reservoir activity. This seems to be corroborating the reports, where in certain legumes such as soybean, pod wall plays an important role in carbohydrate acquisition and storage as sink before they are remobilized and translocated to the developing seeds [32]. Pod shells are the first line of defense against pathogen attack for the seeds, which has been reflected by defense, detoxification and signaling pathway in response to cellular stimuli, plant-pathogen interaction and photo-oxidation. Similarly, based on the GO annotation, metabolic and cellular processes, catalytic activities, binding and cellular components such as cell and membrane were found to be mainly involved during seed development. This result corresponds to those identified in chickpea during seed development revealing higher metabolic and cellular activities in membrane and cell [11].

Seed development is a highly regulated process controlled by various TFs. These TFs may act in a tissue specific manner and play distinct role in tissue development, function and transcriptional regulation [33, 34]. Some other TFs may display stage-specific expression patterns. Few seed-specific TFs were identified in the present study such as B3 DNA binding domain TFs-*ABI3*, *FUS3*, which were known to regulate the process of seed maturation while preventing premature seed germination [34, 35]. Other TFs such as CUP-SHAPED COTYLEDON play important role in cotyledon initiation and development [36]. These TFs could be critical players in determining cell fate and cotyledon differentiation during seed development [34, 37]. In addition, the role of phytohormones such as abscisic acid (ABA), gibberellin and auxins in regulating these processes cannot be undermined [3, 38, 39]. For instance, processes such as embryo patterning, organogenesis, seed maturation requires constant interplay of auxin signaling [39–41].

## Conclusions

The sequencing data generated was comprehensively analyzed for identifying spatio-temporal gene expression, seed specific transcriptional regulators, co-regulated and co-expressed genes. These revealed important metabolic pathways and biological processes involved in the seed development. The dataset would be useful in identifying the candidate genes co-localized in the genomic regions underlying agronomic traits. It would also be useful for understanding the baseline expression, when comparing an interesting mutant for seed related traits. This useful resource could be utilized for functional genomics, candidate gene discovery and genomics-assisted breeding efforts in pigeonpea and related legumes.

## Supporting Information

S1 Fig Pigeonpea samples used for seed and pod wall development study. (TIF)

S2 Fig Validation of RNA-seq data using qPCR. (TIFF)

S1 Table List of constitutively expressed genes. (XLSX)

S2 Table List of pathways identified in KEGG database. (XLSX)

S3 Table Major families of identified transcription factors. (XLSX)

S4 Table Matrix displaying the DEGs identified in different pairwise combinations. (XLSX)

S5 Table List of primers used for qPCR validation. (XLSX)

S6 Table Temporally expressed genes identified in seed samples. (XLSX)

S7 Table Temporally expressed genes identified in pod wall samples. (XLSX)

S8 Table Genes expressed specifically in different samples. (XLSX)
