# Supplementary figures and images for: Deciphering Transcriptional Programming during Pod and Seed Development Using RNA-Seq in Pigeonpea (Cajanus cajan)
Source: PLoS One. 2016 Oct 19;11(10):e0164959. doi: 10.1371/journal.pone.0164959 (PMC5070767; doi:10.1371/journal.pone.0164959)

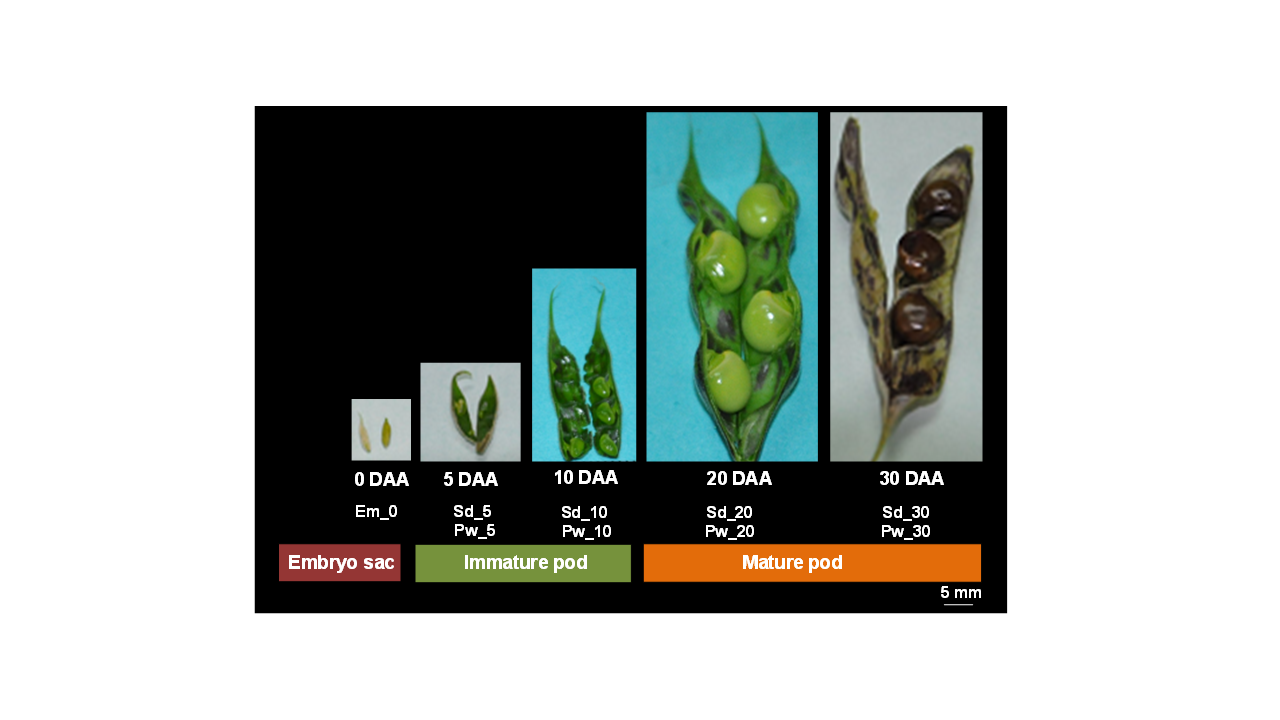

Supplement: S1 Fig — (TIF) [file pone.0164959.s001.tif]
